# Phytosphingosine ceramide mainly localizes in the central layer of the unique lamellar phase of skin lipid model systems

**DOI:** 10.1016/j.jlr.2022.100258

**Published:** 2022-08-02

**Authors:** Andreea Nădăban, Gerrit S. Gooris, Charlotte M. Beddoes, Robert M. Dalgliesh, Joke A. Bouwstra

**Affiliations:** 1 Division of BioTherapeutics, Leiden Academic Centre for Drug Research, Leiden University, Leiden, The Netherlands; 2 ISIS Neutron and Muon Source, Science and Technology Facilities Council, Rutherford Appleton Laboratory, Didcot, United Kingdom

**Keywords:** stratum corneum, long periodicity phase, FTIR, neutron diffraction, sphingolipids, cholesterol, fatty acids, lipid arrangement, lipid organization, inflammatory skin diseases, CER, ceramide, CHOL, cholesterol, DFFA, perdeuterated FFA, EOS, N-(30-linoleoyloxy-triacontanoyl)-sphingosine, FTIR, Fourier-transform infrared spectroscopy, LPP, long periodicity phase, NP, *N*-(tetracosanoyl)-phytosphingosine, NPd47, CER NP with a perdeuterated acyl chain (d47), NS, *N*-(tetracosanoyl)-sphingosine, NSd47, CER NS with a perdeuterated acyl chain (d47), NSd7, CER NS with terminally deuterated sphingosine chain (d7), SANS, small-angle neutron scattering, SC, stratum corneum, SLD, scattering length density, SPP, short periodicity phase

## Abstract

Understanding the lipid arrangement within the skin’s outermost layer, the stratum corneum (SC), is important for advancing knowledge on the skin barrier function. The SC lipid matrix consists of ceramides (CERs), cholesterol, and free fatty acids, which form unique crystalline lamellar phases, referred to as the long periodicity phase (LPP) and short periodicity phases. As the SC lipid composition is complex, lipid model systems that mimic the properties of native SC are used to study the SC lipid organization and molecular arrangement. In previous studies, such lipid models were used to determine the molecular organization in the trilayer structure of the LPP unit cell. The aim of this study was to examine the location of CER *N*-(tetracosanoyl)-phytosphingosine (CER NP) in the unit cell of this lamellar phase and compare its position with CER *N*-(tetracosanoyl)-sphingosine (CER NS). We selected CER NP as it is the most prevalent CER subclass in the human SC, and its location in the LPP is not known. Our neutron diffraction results demonstrate that the acyl chain of CER NP was positioned in the central part of the trilayer structure, with a fraction also present in the outer layers, the same location as determined for the acyl chain of CER NS. In addition, our Fourier transformed infrared spectroscopy results are in agreement with this molecular arrangement, suggesting a linear arrangement for the CER NS and CER NP. These findings provide more detailed insight into the lipid organization in the SC lipid matrix.

## Graphical abstract

**Figure:**
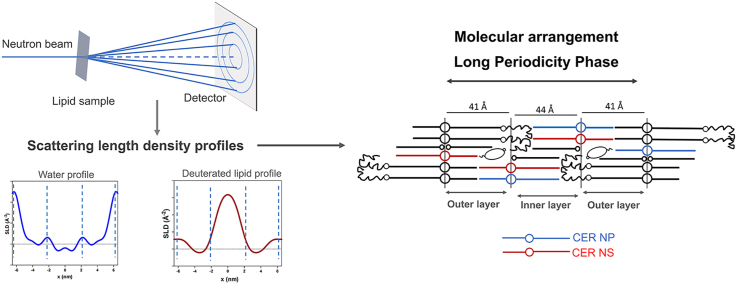

The skin acts as a barrier to protect the body against the environment (1). The skin barrier function is primarily located in the outermost layer of the skin, the stratum corneum (SC), which consists of corneocytes (dead keratin containing cells) embedded in a lipid matrix. The SC lipid matrix provides the only continuous pathway for substances through the SC. This lipid matrix is therefore considered a crucial element in the barrier function (2). The three main SC lipid subclasses are ceramides (CERs), free fatty acids (FFAs), and cholesterol (CHOL), in an approximately equimolar ratio (3, 4, 5). The CER subclasses differ by their molecular structure, as they consist of a long acyl chain linked through an amide bond to a sphingoid base (6, 7, 8). The CER nomenclature used is according to Motta *et al.* (9). Nowadays at least 21 different CER subclasses have been identified in human SC (8, 10, 11).

X-ray diffraction studies revealed that the SC lipids simultaneously form two unique crystalline lamellar phases referred to as the short periodicity phase (SPP) and long periodicity phase (LPP), with a repeat distance of approximatively 6 and 13 nm, respectively (12, 13). Apart from this lamellar organization, the lateral organization of the lipids is also important for barrier functionality (14). The lateral packing represents the arrangement of the lipids within the lamellae. The lipids are packed in either an orthorhombic (densely packed lipids), a hexagonal (less dense packing, but still an ordered organization), or a liquid (fluid) (disordered lipids) phase. In human SC, the lipids primarily adopt an orthorhombic packing at physiological temperature, while a small portion of the lipids adopt a hexagonal packing (14, 15, 16). In lipid model membranes, besides the hexagonal and orthorhombic packing, a liquid or isotropic phase has also been encountered (17, 18, 19).

When focusing on CER composition, changes in CER subclass composition have been reported in several inflammatory skin diseases, such as atopic dermatitis, psoriasis, or Netherton syndrome, which correlated with an impaired skin barrier function, demonstrating that the lipid composition is important for the skin barrier (9, 10, 20, 21, 22, 23, 24). Clinical studies revealed that especially the concentration of CER *N*-(tetracosanoyl)-sphingosine (CER NS) was increased, while the concentration of CER *N*-(tetracosanoyl)-phytosphingosine (CER NP) was reduced (Fig. 1) (9, 24, 25, 26). Furthermore, another interesting observation can be made when comparing the CER subclass composition in porcine, dog, mice, and human SC. In dog, mice, and porcine SC, CER NS is by far the most abundant CER subclass (11, 27, 28, 29), while in healthy human SC, CER NP is one of the most abundant CER subclasses (6, 8, 30). Therefore, a comparison of the role of CER NP and CER NS in the lipid organization will result in a more thorough understanding of the lipid arrangement and the barrier function. In native SC it is not possible to study selectively the role of these CER subclasses; thus, for such studies lipid model systems are an attractive tool.

**Fig. 1:**
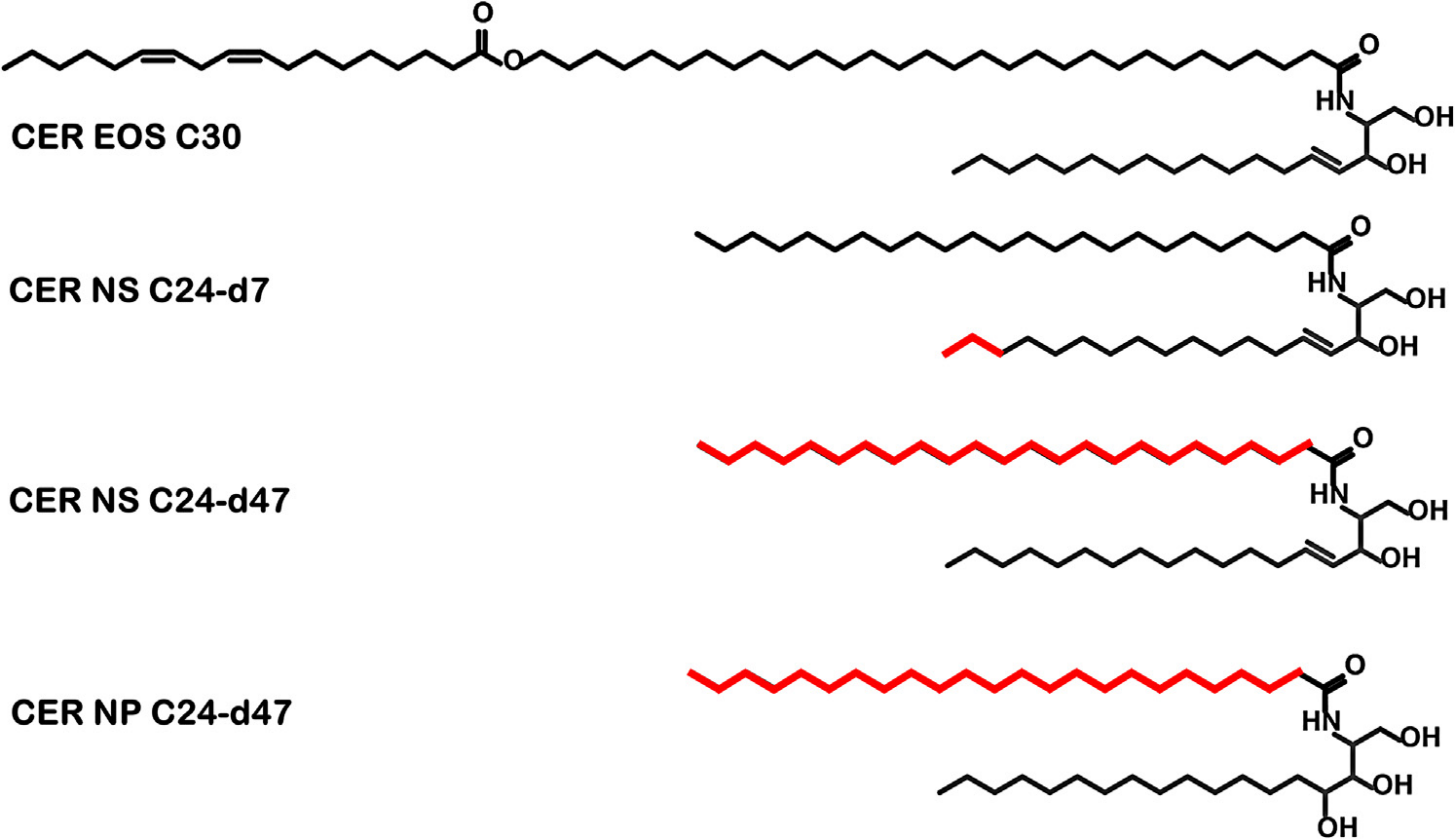
The molecular structure of the CERs used in this study. The deuterated chains are highlighted in red: the d7 sphingosine chain of CER NS and the acyl chains d47 of CER NS and CER NP.

Lipid model membranes can be used as an alternative to investigate the relationship between lipid composition, lipid organization, and lipid barrier functionality. These systems are prepared with SC lipids, using selected CER subclasses, CHOL, and a suitable FFA chain length composition, creating a model that resembles the lipid composition and organization in healthy SC lipid matrix (17, 21, 29, 31, 32, 33, 34).

Previous studies have shown the importance of the lamellar lipid organization for maintaining the skin barrier functionality, in which the presence of the LPP plays an important role (23, 35). Using lipid models, it was identified that CER *N*-(30-linoleoyloxy-triacontanoyl)-sphingosine (CER EOS) is required for the formation of the LPP (36); therefore, including CER EOS in the lipid composition is essential for the SC lipid matrix (34, 37, 38, 39). The unit cell of the LPP consists of three stacked lipid layers, as illustrated by the electron density profile and water profiles determined by analyzing the X-ray diffraction pattern (including that of human SC) and neutron scattering profiles, respectively (32, 40). Investigating the molecular organization of the lipids within the LPP trilayer unit is important for understanding the role of the different lipid subclasses in the formation of this phase. To achieve this, it is important that the lipid mixture only forms the LPP. This can be obtained by increasing the CER EOS level to at least 30 mol% of the total amount of CERs without changing the unit cell structure (41, 42).

Small-angle neutron scattering (SANS) is an excellent tool that can be used to investigate the localization of lipid subclasses (40, 43, 44, 45, 46). Previous studies focused on the localization of CER NS, CER EOS, CHOL, and FFA in the LPP, and based on these results a trilayer unit molecular arrangement was proposed (44). However, to our knowledge the location of CER NP, the most abundant CER in the human SC in the LPP, is not yet determined. Therefore, the localization and arrangement of CER NP in the LPP is the aim of the present study.

We have investigated the position of the acyl chain of CER NP in the LPP unit cell using SANS and compared it with the acyl and sphingosine chains of CER NS. The lipid model consisted of only five lipids, CER EOS, CER NS, CER NP, CHOL, and FFA C24 (chain length 24 carbon atoms). This allows a more detailed analysis especially as Fourier transformed infrared (FTIR) spectroscopy is used to assess the lateral organization and the lipid chain interactions. As we are primarily interested whether the head group architecture affects the position and arrangement of the CERs, we chose an equal fraction of CER NP and CER NS. The location of CER NP was determined and compared with that of CER NS, using CER NP and CER NS with deuterated acyl chains. First, we showed that this composition indeed formed the LPP, as observed in more complex lipid model systems and intact SC. Within the LPP, CER NP adopts a similar localization as CER NS, with the acyl chains of CER NP being localized predominantly in the inner layer of the LPP unit. Furthermore, we demonstrate that the acyl chains of CER NP and CER NS and the FFA C24 chains are neighbors in the central layer of the LPP unit cell and that CER NP adopts a linear conformation, similar to that of CER NS.

## Materials and methods

### Materials

The synthetic CER mixture used for the LPP model consists of CER EOS, CER NS, and CER NP, whose molecular structures are presented in Fig. 1. The sphingoid chains of CER NS and CER NP have a chain length of 18 carbons (C18), while the acyl chain length is 24 carbons (C24). These protiated CERs, alongside the deuterated CER NS and CER NP with a perdeuterated acyl chain (d47), referred to as NSd47 and NPd47, respectively, were kindly provided by Evonik (Essen, Germany). CER NS with the sphingosine chain terminally deuterated, referred to as NSd7, was purchased from Avanti Polar Lipids (Alabama). Lignoceric acid (FFA24), CHOL, acetate buffer salts, and D_2_O were supplied by Sigma-Aldrich Chemie GmbH (Schnelldorf, Germany). Deuterated lignoceric acid (DFFA24) was obtained from Arc Laboratories BV (Apeldoorn, The Netherlands). All organic solvents were HPLC grade or higher and were purchased from Biosolve BV (Valkenswaard, The Netherlands). The Millipore quality water was produced by a Milli-Q water filtration system. The silicon substrates (wafers) were purchased from Okmetic (Vantaa, Finland).

### Composition of the lipid models

The lipid models were prepared from the synthetic CERs, CHOL, and FFA in a 1:1:1 molar ratio. In order to form exclusively the LPP, the ratio of CER EOS was set to 40 mol% of the total CER concentration (41). The concentrations of CER NS and CER NP were equal, each 30 mol% of the total CERs. In this study the FFA included was only FFA24, in order to limit the total number of components in the model. The composition of the lipid models with their molar ratios and the abbreviations are provided in Table 1.

**Table 1:** The composition of the lipid model membranes and the molar ratios

Model Abbreviation	Lipid Composition	Molar Ratio
LPP:prot	CER EOS: CER NS: CER NP: CHOL: FFA C24	0.4:0.3:0.3:1:1
LPP:NPd47	CER EOS: CER NS: **CER NPd47**: CHOL: FFA C24	0.4:0.3:**0.3**:1:1
LPP:NSd47	CER EOS: **CER NSd47**: CER NP: CHOL: FFA C24	0.4:**0.3**:0.3:1:1
LPP:NSd7	CER EOS: **CER NSd7**: CER NP: CHOL: FFA C24	0.4:**0.3**:0.3:1:1
LPP:NSd47:DFFA24	CER EOS: **CER NSd47**: CER NP: CHOL: **DFFA C24**	0.4:**0.3**:0.3:1:**1**
LPP:NPd47:DFFA24	CER EOS: CER NS: **CER NPd47**: CHOL: **DFFA C24**	0.4:0.3:**0.3:**1:**1**
LPP:NSd47:NPd47:DFFA24	CER EOS: **CER NSd47**: **CER NPd47**: CHOL: **DFFA C24**	0.4:**0.3:0.3**:1:**1**

The deuterated lipids and their molar ratios are highlighted in bold.

### Sample preparation of the lipid models

To prepare the samples, the required amount of lipids was dissolved in a chloroform/methanol solution (2:1; v/v) at a concentration of 5 mg/ml. The samples were sprayed using a Camag Linomat IV device (Muttenz, Switzerland) at a rate of 14 s/μl, under a gentle stream of nitrogen. For the FTIR measurements, 1 mg of the lipid mixture was sprayed on AgBr windows over an area of 1 x 1 cm^2^. The lipids were then equilibrated by increasing the temperature to 85°C (using a constant rate of 4°C/min), and it was maintained at this temperature for 50 min to ensure the lipids melted. This was followed by a slow cooling to room temperature. For the neutron diffraction experiments, 10 mg of lipids was sprayed on a silicon substrate in an area of 1.2 × 3.8 cm^2^ using the sample preparation method described above. To avoid contraction of the lipid layers during the melting process, the sample equilibration temperature was 81–82°C. Prior to the measurements, the samples were hydrated either with deuterated acetate buffer (pH 5.0) (for FTIR studies) or a D_2_O/H_2_O buffer (for neutron experiments) at 37°C for ≥12 h. For contrast variation in the neutron experiments, the samples were hydrated at three different D_2_O/H_2_O buffer levels (100%, 50%, and 8%).

### Neutron diffraction measurements

The neutron diffraction data were collected at the ISIS Neutron and Muon Source (Rutherford Appleton Laboratory, United Kingdom), on the LARMOR instrument, set in SANS mode. The wavelength range of the neutron beam was 1–12.5 Å, and its diameter was 1 × 30 mm. The distance from the sample to the detector was 4.4 m. The ^3^He tube detector angle was set at a 2θ angle of 5° to the direct neutron beam and covered approximately 4° in both directions. The incident flux shape and detector efficiency were accounted for by using the direct beam measurement; thus, all the different curves measured at the different wavelengths would overlap. Each sample sprayed on the silicon substrate was placed in a custom-made aluminum humidity chamber maintained at a constant temperature of 25°C and measured for 4 h (40 μA/h accelerator proton charge). The windows of the aluminum chambers were maintained at 42°C to prevent condensation. The sample angle to the neutron beam was set to 2.5°, and kinematic mounts ensured reproducible positions. An empty aluminum chamber was measured as background and was subtracted from each scattering curve.

### Neutron data analysis

The MANTID software framework was used for the SANS data analysis (47). First, the detector readout was reduced to one-dimensional plots and the background (empty chamber measurement) was subtracted. The data were then analyzed following the steps described previously (40). Briefly, a one-dimensional diffraction pattern of the scattering intensity as a function of the scattering angle (2θ) was obtained. The 2θ scattering angle was converted to the scattering vector *q* using the following equation:

(1) $$q=\frac{4\pi sin\theta }{\lambda }$$

where *λ* is the wavelength of the neutron beam and θ is the Bragg angle. The repeat distance of the LPP unit cell (*d*) was calculated from the position of a series of equidistant diffraction peaks attributed to the lamellar phase (Bragg peaks):

(2) $$d=\frac{2\pi n}{q_{n}}$$

where n is the order of the diffraction peak. To obtain the intensity (I_n_) of each peak, the Bragg peaks were fitted using a Pearson VII function (using Fityk software). From these peak intensities the absolute structure factor amplitude for each diffraction order (|F_n_|) was calculated:

(3) $$|F_{n}|=A_{n}\sqrt{LI{\;}_{n}}\;$$

In this formula, *L* refers to the Lorentz correction and it can be assumed *L = q* because of the high degree of orientation of the lipid lamellae in the samples. *A*_*n*_ represents the correction factor for the sample absorption, which can be calculated using the following equation (48):

(4) $$A_{n}=\frac{1}{\sqrt{\frac{sin\theta }{2\mu l}\left(1-e^{\frac{-2\mu l}{sin\theta }}\right)}}$$

where *μ* is the linear attenuation coefficient and *l* represents the lipid film thickness. The latter was calculated to be ∼0.03 mm, knowing the lipid density and surface area of the sample, as described previously (49).

The D_2_O/H_2_O contrast variation method was used to determine the water phase signs of the amplitudes of the different diffraction orders. The lipid head groups are located at the boundary of the lamellar phases (40, 49, 50). In a hydrated lipid model, most of the water molecules are located close to the hydrophilic lipid head groups, rather than the hydrophobic tails. With this assumption, the phase signs of the water profile were selected, which can be either positive (+) or negative (-). The phase signs of the water profile are obtained from the positive or negative signs of the slope of the difference between the absolute structure factors |F_n_| of the sample hydrated at 100% and 8% D_2_O/H_2_O. The combination of phase signs for the water profiles in this study is - + - + - +. Using these phase signs, four regions are observed in the LPP unit cell, corresponding to the lipid head group locations at the boundary of the unit cell and in the inner layer. Other phase sign combinations resulted in unrealistic water profiles and were thus discarded. The phase signs of the water profile used in this study are in agreement with previously reported structure factor phase signs for the LPP (32, 40, 44, 46).

**Fig. 2:**
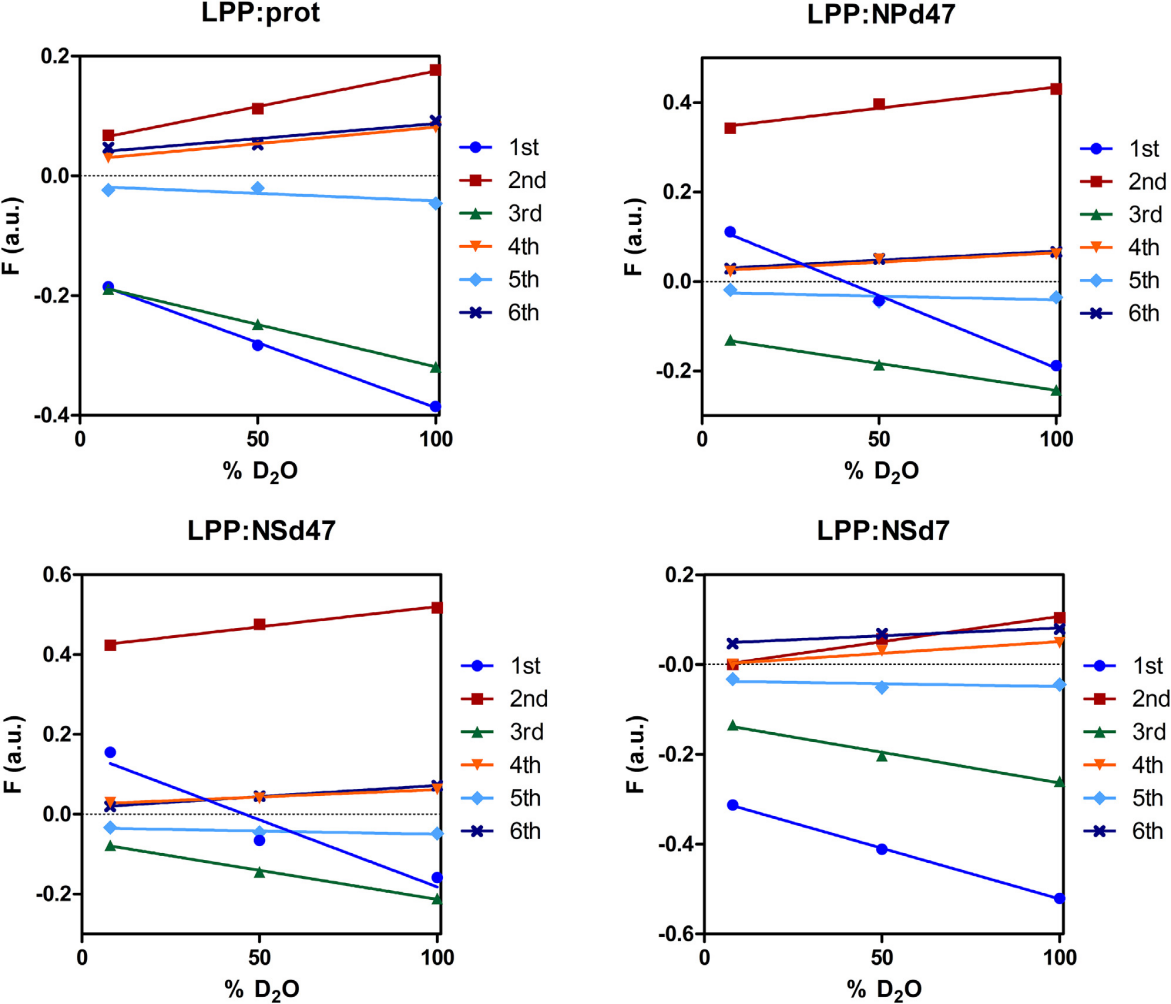
Linear fitting of the relative structure factors (F) as a function of the percentage of D_2_O in the D_2_O/H_2_O buffer for the six diffraction orders of the LPP:prot, LPP:NPd47, LPP:NSd47, and LPP:NSd7 models. The diffraction orders are each represented by different colors and symbols: first (dot, dark blue), second (square, red), third (triangle, green), fourth (triangle, orange), fifth (diamond, light blue), sixth (cross, purple).

Next, the structure factors (calculated with Equation 3) with the corresponding phase signs are plotted as a function of the D_2_O/H_2_O buffer ratio (Fig. 2). There is a linear correlation of the relative structure factor amplitudes as a function of the D_2_O/H_2_O buffer ratio, thus demonstrating the centrosymmetric structure of the LPP unit cell. This is in agreement with previous studies (40, 44). The phase signs of the protiated and deuterated samples were individually determined based on the positive or negative sign of the structure factors at 8% D_2_O/H_2_O hydration (Fig. 2). For the LPP:prot, and LPP:NSd7 lipid models, the same combination of phase signs was obtained (- + - + - +). The phase signs of the samples match those obtained for the water profile if the linear regression of the structure factors plotted as a function of the buffer level does not intersect the *x*-axis. The phase sign is switched for the respective diffraction order if the line crosses the *x*-axis. This can be seen in the LPP:NPd47 and LPP:NSd47 models (Fig. 2) for the first diffraction order, when the slope of the regression line is negative but the F_n_ at 8% D_2_O/H_2_O has a positive value, determining the phase signs combination + + - + - +.

Using the phase signs and the values of the structure factors, the scattering length density (SLD) profile of the unit cell was calculated by Fourier reconstructions:

(5) $$\rho \left(x\right)=F_{0}+2\overset{n_{max}}{\underset{n=1}{\sum }}F_{h}\;cos\left(\frac{2\pi nx}{d}\right)$$

where *x* is the distance in the unit cell, with *x*=0 center of the unit cell (40). *F*_*0*_, the scattering density per unit volume, was calculated using the chemical composition of the lipid sample (which included one water molecule per lipid molecule from the hydration of the samples) and its density (51) https://www.ncnr.nist.gov/resources/activation/ (Accessed: 2021).

The SLD profiles of each deuterated lipid moiety were calculated by subtracting the protiated profiles from the deuterated profiles of each sample hydrated at 8% D_2_O/H_2_O buffer. At this exact buffer ratio, the scattering contribution of the buffer is zero and the SLD profiles show only the scattering of the lipid chains.

A scaling factor was calculated to place the SLD data on a “relative absolute” scale, as previously described (36, 44, 52). First, the SLD peak height (SLD_h_) and peak area (SLD_a_) were fitted for the sample with NSd7. The peak area obtained from the difference in SLD profile (SLD_dif_) of the LPP:NSd7 and LPP:prot lipid models represents the deuterium atoms on the CER sphingosine chain. The relative absolute SLD value (SLD_correct_) was determined using the following equation:

(6) $$SLD_{correct}=\frac{SLD_{h}*SLD_{dif}}{SLD_{a}}\;$$

Finally, the scaling factor was calculated as the ratio between SLD_correct_ and SLD_h_ and it was then applied to the F_n_ values to obtain the results on the relative absolute scale.

### FTIR measurements

The FTIR measurements were performed on a Varian 670-IR spectrometer (Agilent Technologies, Santa Clara) using a broad-band mercury cadmium telluride detector, cooled by liquid nitrogen. The FTIR spectra were acquired by the coaddition of 256 scans, with a resolution of 1 cm^−1^, collected over 4 min in transmission mode. Starting 30 min prior to the measurement, the samples were purged continuously under dry air. In order to examine the thermotropic phase behavior, the lipid models were measured between 10 and 90°C at a heating rate of 0.25°C/min (resulting in a 1°C temperature rise per recorded spectrum). The measurement was performed in the wavenumber range of 600–4,000 cm^−1^. The software Resolution Pro (Agilent Technologies, Palo Alto) was used for data collection and analysis. During data analysis, the spectra were deconvoluted using a half-width of 4 cm^−1^ and an enhancement factor of 1.4. At least three samples were prepared and measured for each experimental condition.

The conformational ordering determined at 10°C and phase transitions of the lipid chains were examined using the CH_2_ symmetric stretching vibration (ν_s_CH_2_, wavenumber range: 2,845-2,855 cm^−1^) and CD_2_ symmetric stretching vibration (ν_s_CD_2_, 2,080-2,100 cm^−1^). The lateral packing of the lipids was analyzed using the CH_2_ scissoring vibration (δCH_2_, 1,462-1,473 cm^−1^), while the mixing properties of the lipid chains was determined using the CD_2_ and CH_2_ scissoring vibration (δCD_2_, 1,085-1,095 cm^−1^). The accurate peak position determination of the δCH_2_ and δCD_2_ vibrations was performed in the Enthought Canopy software, using in-house developed Python scripts. The three scissoring peaks were fitted using a Lorentzian function.

Statistical analysis was performed using GraphPad Prism (v.8). An unpaired *t* test was conducted to determine the significance of the peak height ratio values of the two orthorhombic δCD_2_ modes (at 1,086 and 1,091 cm^-1^) and the central δCD_2_ peak (at 1,088.5 cm^-1^). Differences in mean values of the different models (n ≥ 3) are considered statistically significant when *P* < 0.05.

The mid-point temperature of the ordered-disordered phase transition (T_M_) was determined by fitting a linear regression curve, as described before (53).

## Results

### Localization of CER NP and CER NS in the LPP lipid model system

**Fig. 3:**
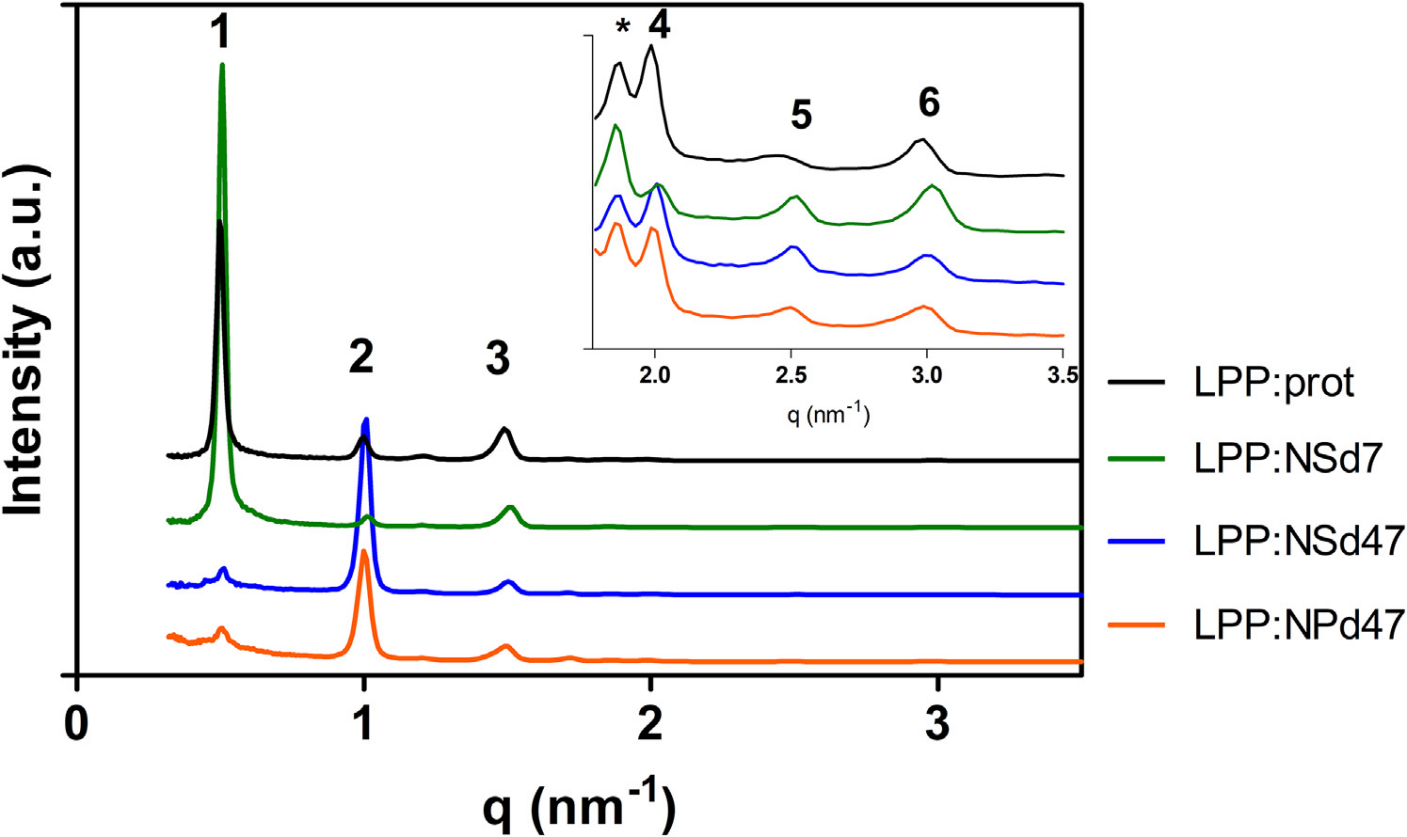
The one-dimensional neutron diffraction patterns of the four samples hydrated at 100% D_2_O/H_2_O (LPP:prot, LPP:NPd47, LPP:NSd47, and LPP:NSd7), the intensity plotted as a function of the scattering vector (q). The inset represents the zoomed-in region q=1.7–3.5 nm^−1^. The first six diffraction orders are indicated by numbers and the crystalline CHOL peak is indicated by an asterisk.

Neutron diffraction studies were performed to examine the molecular arrangement of CER NP and CER NS in the LPP trilayer unit. In Fig. 3 the neutron patterns of the lipid samples are depicted and they showed six equidistant peaks that all could be attributed to the LPP. A small peak appeared at q=1.2 nm^-1^, which did not overlap the Bragg peaks corresponding to the LPP, indicating that a very small portion of the lipids formed another phase. The reflection attributed to crystalline phase separated CHOL was observed at q=1.8 nm^−1^, and it did not interfere with the diffraction orders of the LPP. The repeat distance of the LPP unit was calculated from the series of equidistant Bragg peaks with Equation 2. The d-spacing values obtained for the four samples were very similar: 12.6 ± 0.1 nm (LPP:prot), 12.5 ± 0.05 nm (LPP:NSd7), 12.5 ± 0.1 nm (LPP:NSd47), and 12.6 ± 0.1 nm (LPP:NPd47).

**Fig. 4:**
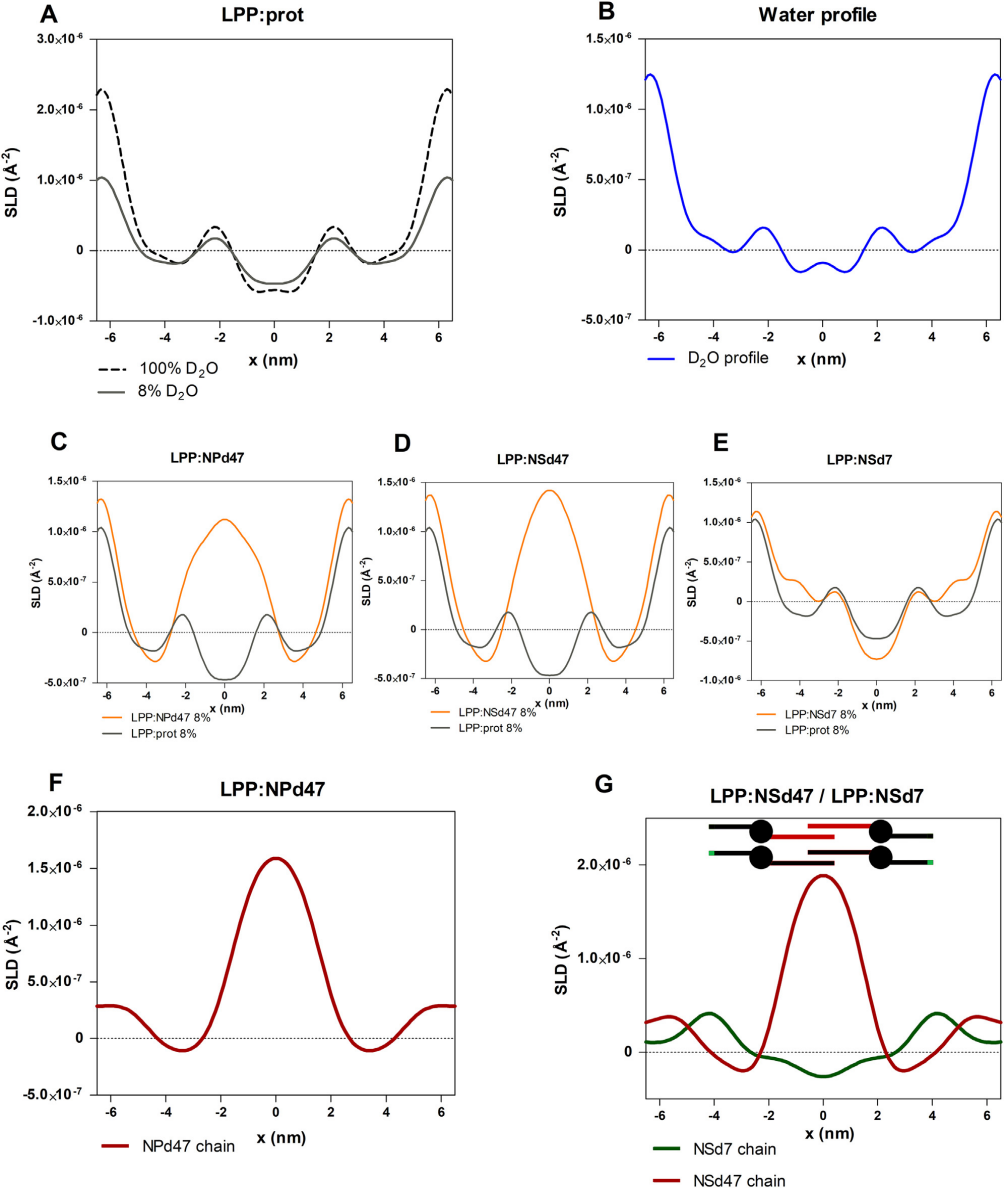
Neutron SLD profiles of (A) LPP:prot, measured at 100% (top dashed line) and 8% (bottom solid line) D_2_O/H_2_O hydration; (B) the D_2_O (water) profile determined from the difference between the 100% and 8% SLD profiles of the LPP:prot model; (C) LPP:prot (gray line) and LPP:NPd47 (orange) overlapped, both measured at 8% D_2_O/H_2_O buffer ratio; the difference between these represents the net SLD profile of the deuterated moiety; (D) LPP:prot (gray) and LPP:NSd47 (orange) samples, at 8% D_2_O/H_2_O hydration; (E) LPP:prot (gray) and LPP:NSd7 (orange) models, measured at 8% D_2_O/H_2_O; (F) The net SLD profile of the perdeuterated CER NPd47 chain (red line), determined from the SLD profile of LPP:prot subtracted from the LPP:NPd47 model; (G) the net SLD profile of the deuterated acyl chain of CER NSd47 (red line) and sphingosine chain of CER NSd7 (green line) obtained from the two models: LPP:NSd47 and LPP:NSd7. The maximum SLD intensity for the NSd47 chain was predominantly localized in the inner layer of the LPP unit, but with a small part also in the outer layers, while NSd7 was located in the outer layers of the LPP.

Data analysis based on the calculated structure factors and phase signs led to the construction of the SLD profiles, using Equation 5. Figure 4A displays the SLD profile of the LPP:prot sample hydrated at 100% and 8% D_2_O/H_2_O. The intensity of the SLD profiles is directly proportional to the percentage of D_2_O in the hydration buffer; thus, the highest intensity was noticed after hydration with 100% D_2_O/H_2_O, while the SLD profile of the sample after 8% D_2_O/H_2_O hydration has no contribution of the water (supplemental Fig. S1A). Therefore, by subtracting the profile of the 8% D_2_O/H_2_O from the 100% D_2_O/H_2_O buffer, the water profile for this sample is obtained. This SLD water profile is shown in Fig. 4B, and it indicates that the water molecules were present at the outer regions of the centrosymmetric LPP unit cell (at 6.3 ± 0.1 nm from the center) and at two distinct regions inside the unit cell (2.2 ± 0.1 nm from the unit cell center). The positions of the water profile correspond to the locations of the hydrophilic head groups of the lipids. Therefore, the SLD water profile is in agreement with the trilayer structure of the LPP (40, 44). The water profile followed the same pattern in all four compositions analyzed in this study.

Using the phase signs for the deuterated samples, the next step was to calculate the SLD profiles for the LPP:NPd47, LPP:NSd47, and LPP:NSd7 compositions from the structure factors for each of the three D_2_O/H_2_O buffer hydration levels (supplemental Fig. S1). Then the 8% D_2_O/H_2_O hydrated samples were selected for calculating the localization of the deuterated samples, as at this ratio there is no contribution of water to the SLD profile. The SLD profiles are provided in Fig. 4C–E. To identify the localization for the deuterated lipid chains in these systems, the difference between the SLD profile of the deuterated and the protiated model was determined. The difference of the SLD profiles indicates the location of the deuterated moiety in the LPP trilayer unit. Figure 4F clearly shows that the maximum of the SLD profile was obtained in the inner layer of the LPP unit; however, there is an SLD elevation at the unit cell border. This indicates that the perdeuterated acyl chain of CER NP is primarily located in this inner layer of the LPP unit, with a small fraction of the chains positioned in the outer layers.

Similarly, the SLD profiles of the deuterated chains of CER NS were calculated in the LPP:NSd47 and LPP:NSd7 models and are both shown in Fig. 4G. The SLD profile indicates that the maximum intensity of the d47 acyl chain of CER NS was located in the central layer of the LPP trilayer unit cell but an increase in SLD profile was also observed near the LPP unit cell border (Fig. 4G, red curve). Therefore, the deuterated acyl chains of CER NS were localized predominantly in the inner layer of the LPP, but a fraction of the CER NS acyl chains is also positioned in the outer layers. This indicates that both the CER NS and CER NP head groups are located not only at the inner water layer region but also at the unit cell border, although clearly less abundant.

CERs can adopt two conformational arrangements: in an extended (linear) conformation the lipid tails are located on either side of the lipid headgroup, while in a hairpin conformation the two lipid chains are located on the same side of the headgroup. In this study, the conformation of the CER NS was examined by determining the localization of the terminally deuterated sphingosine chain of CER NSd7. The SLD profile of the d7-deuterated sphingosine chain of CER NS (LPP:NSd7) presented in Fig. 4G (green curve) clearly revealed that the maximum scattering intensity was located at 4.2 nm from the center of the LPP unit cell (respectively, 2 nm outer unit cell boundary in the two outer lipid layers). No maximum was observed in the inner layer. This demonstrates that the CER NS with the head groups in the inner head group regions have their acyl chain in the central lipid layer and the sphingosine tail in the outer lipid layers. Therefore, these lipids are in a linear arrangement. When CER NS is either in a linear or hairpin conformation in the outer layers, the deuterated sphingosine moiety will be located at a very similar position coinciding to that of the CER NS located in the inner layers of the LPP. Therefore, we were unable to determine the conformation of the CER NS with the head group located at the unit cell boundaries of the LPP.

### ν_s_CH_2_ frequencies indicate mixing of the lipids

**Fig. 5:**
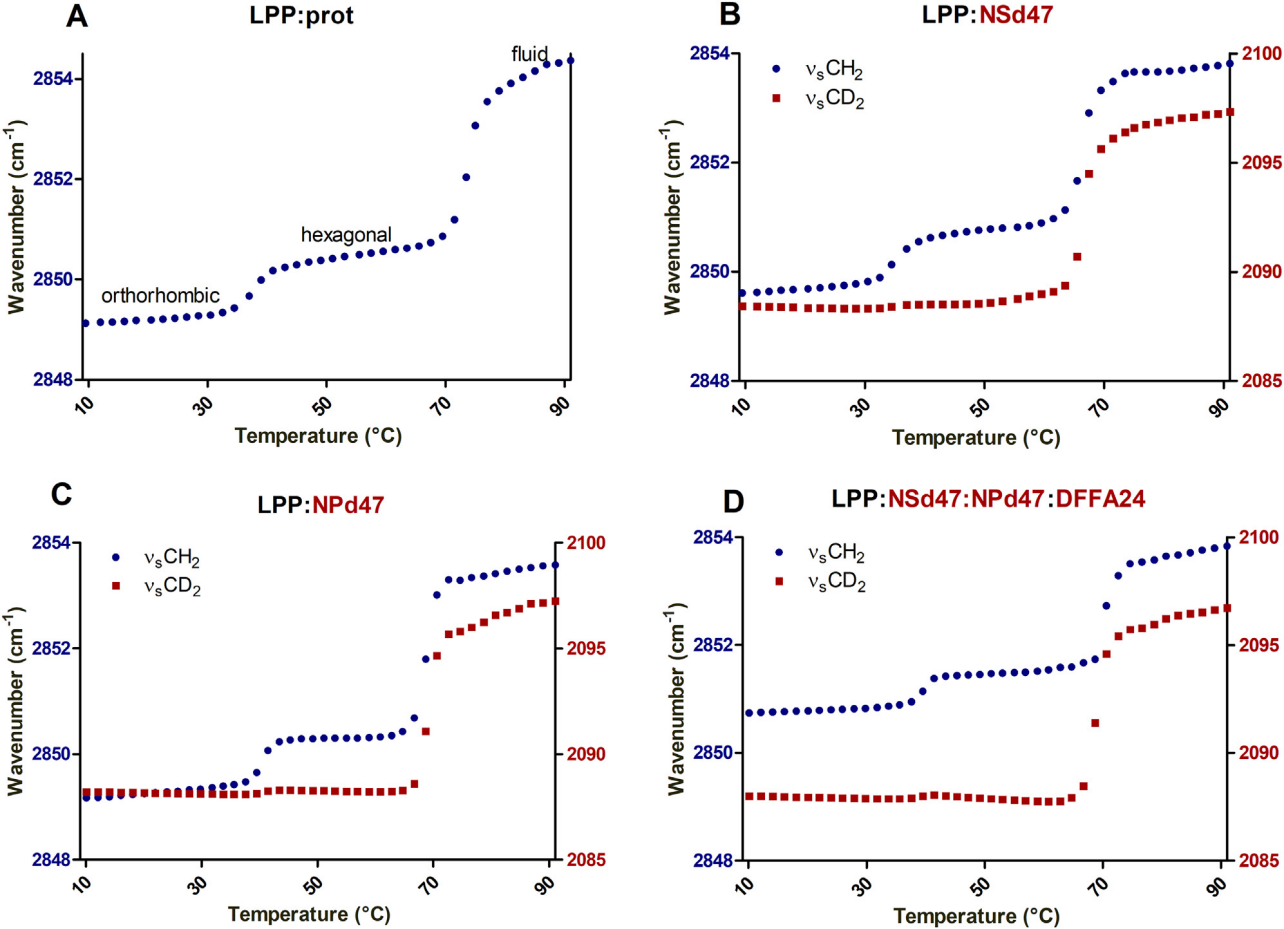
Thermotropic curves of the stretching vibrations for the LPP:prot lipid model (A) and deuterated models with CER NSd47(B) and CER NPd47(C) and the LPP model with CER NSd47, CER NPd47, and DFFA24 (D). The phase transitions temperatures of the lipids are plotted as a function of the ν_s_CH_2_ and ν_s_CD_2_ peak position, on the left and right *y*-axis, respectively. Both the protiated (blue circle) and deuterated (red square) lipids melted over the same temperature range, indicating that the deuterated lipids were integrated with the protiated lipids in the model membrane.

Information about the thermotropic behavior and the lipid packing is obtained from the ν_s_CH_2_ peak position in the FTIR spectra. The ν_s_CH_2_ vibrations are a measure for the conformational disordering of the hydrocarbon chains. Based on the ν_s_CH_2_ wavenumber, the transitions from an ordered to a disordered lipid model can be monitored. Figure 5A depicts the thermotropic behavior of the LPP:prot sample. The ν_s_CH_2_ vibrations indicate that between 10°C and 35°C the lipid chains of the LPP:prot model were organized in an ordered phase (ν_s_CH_2_ peak position at 10°C: 2,849 cm^−1^). Between 35 and 40°C a transition from the orthorhombic to the hexagonal phase occurred. This is monitored by an increase of the ν_s_CH_2_ peak wavenumber with ∼1 cm^-1^. A further increase of the temperature of the sample resulted in a gradual increase of the wavenumber, and at around 69°C the phase transition from the ordered to the disordered fluid phase started. During this transition the ν_s_CH_2_ wavenumber increased from 2,850.5 cm^−1^ to 2,854 cm^−1^.

The presence of phase separation in the lipid samples was examined by systematically replacing protiated lipids by their deuterated counterparts (CER NSd47, CER NPd47, and DFFA24). The ν_s_CH_2_ and ν_s_CD_2_ thermotropic responses of the single replacements of the CERs are provided in Fig. 5 B, C. The shifts in the wavenumber of the ν_s_CD_2_ and the ν_s_CH_2_ vibrations indicate a hexagonal to liquid phase transition in a similar temperature range in the LPP:NSd47 and LPP:NPd47 samples, between 65 and 70°C. A similar temperature range for the phase transitions is a first indication that the deuterated and protiated lipids mixed in the same lattice. In the ν_s_CD_2_ vibrations, the orthorhombic to hexagonal transition is hardly visible due to the shift in the ν_s_CD_2_ frequency being less sensitive to this phase transition (54).

When including deuterated DFFA24 alongside either NSd47 or NPd47 in the LPP model, the wavenumber ν_s_CH_2_ peak at 10°C was increased to ∼2,850.5 cm^−1^ for both compositions (supplemental Fig. S2), indicating a higher conformational disordering of the remaining protiated lipids. A similar effect was also observed when FFA and both the CER NS and CER NP were replaced by their deuterated counterparts (LPP:NSd47:NPd47:DFFA24, Fig. 5D) with a further increase to ∼2,850.8 cm^−1^. This wavenumber increase of the ν_s_CH_2_ peak position indicates that the acyl chain of CER EOS with the protiated sphingoid base of CER NS and CER NP had a higher mobility than the acyl chains of CER NS, CER NP, and the DFFA24. Moreover, the shift of the ν_s_CH_2_ wavenumber at 32–34°C indicates an orthorhombic to hexagonal phase transition, which means that a part of the remaining protiated lipids are in an orthorhombic packing, despite the higher wavenumber. This increase in wavenumber is caused by the linoleate chain of CER EOS that represents approximately 19% of the total protiated fraction of hydrocarbon chains and it was previously reported to be in a liquid phase (19). Table 2 displays the ν_s_CH_2_ and ν_s_CD_2_ peak wavenumber at 10°C and the mid-transition temperatures from an ordered to a disordered phase.

**Table 2:** The wavenumber corresponding to the ν_s_CH_2_ and ν_s_CD_2_ peak positions at 10°C, while the lipids were organized in an orthorhombic packing (shown as the mean value of n ≥ 3 measurements, SD of ± 0.1 cm^-1^) and the mid-point transition temperatures (T_M_) for the ordered-disordered (hexagonal-liquid) phase transitions (average ± SD, n ≥ 3)

Lipid Model	νCH_2_ Wavenumber (cm^−1^)	νCD_2_ Wavenumber (cm^−1^)	T_M_ Ordered-disordered Phase Transition (°C)
LPP:prot	2,849.0	-	71.2 ± 1.2
LPP:NSd47	2,849.4	2,088.3	71.4 ± 1.0
LPP:NPd47	2,849.1	2,088.3	68.0 ± 1.5
LPP:NSd47:DFFA24	2,850.4	2,088.1	66.9 ± 1.5
LPP:NPd47:DFFA24	2,850.5	2,088.1	68.5 ± 1.7
LPP:NSd47:NPd47:DFFA24	2,850.8	2,088.0	69.2 ± 3.3

### CD_2_-CD_2_ chain interactions provide information about the CER arrangement

**Fig. 6:**
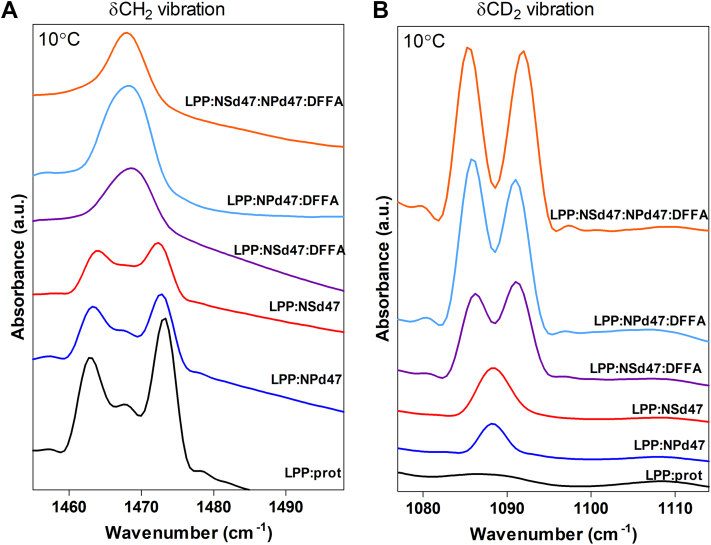
The FTIR peak splitting of the (A) δCH_2_ and (B) δCD_2_ vibrations for the six LPP model membranes, measured at 10°C, when the lipids were packed in the orthorhombic phase. Each curve has an annotation with the lipid model it represents. The peak splitting of the δCH_2_ vibrations amounts to 10.3 ± 0.1 cm^−1^ (LPP:prot), 9.5 ± 0.1 cm^−1^ (LPP:NPd47), and 9.3 ± 0.1 cm^−1^ (LPP:NSd47), while for the δCD_2_ vibrations 5.4 ± 0.1 cm^−1^ (LPP:NPd47:DFFA24), 5.2 ± 0.1 cm^−1^ (LPP:NSd47:DFFA24), and 6.4 ± 0.1 cm^−1^ (LPP:NSd47:NPd47:DFFA24). These values represent the mean ± SD for n ≥ 3 measurements.

To investigate the packing and mixing of the lipid chains in more details, the shape and splitting of the δCH_2_ and δCD_2_ frequencies were examined. The δCH_2_ vibrations of the LPP:prot sample at 10°C is presented in Fig. 6A (black line). Two peaks were observed at 1,463 and 1,473 cm^−1^ with a deep minima in between, an indication that most of the lipid chains were densely organized in an orthorhombic packing, while the small peak at 1,467 cm^−1^ represented the hexagonal packing adopted by a fraction of the lipids. The splitting distance between the two δCH_2_ peaks was calculated, and for the LPP:prot model this distance was 10.3 ± 0.1 cm^−1^. A comparison of this with pure FFA C24, with a maximum δCH_2_ peak splitting of 10.7 cm^−1^ (not shown), provides an indication that the size of the domains forming an orthorhombic phase is close to 100 lipid molecules (55, 56).

In partially deuterated lipid samples, the δCH_2_ and δCD_2_ vibrations of the hydrocarbon chains cannot directly interact due to the large vibrational energy difference (CH_2_: ∼1,470 cm^−1^; CD_2_: ∼1,090 cm^−1^). If the deuterated lipid chains are neighboring one another in large domains, the minima between the two δCD_2_ peaks would be very deep due to the large number of CD_2_-CD_2_ interactions (pure DFFA24 has the maximum splitting distance of 7.3 ± 0.1 cm^−1^, representing a deuterated chain domain size of at least 100 lipid molecules). A decrease in the δCD_2_ splitting distance represents a smaller deuterated domain size. However, if the protiated and deuterated lipid chains are fully integrated and participate in the same orthorhombic lattice, the interaction will be lost and only one peak will be observed. The δCH_2_ and δCD_2_ vibrations in the infrared spectrum of the lipid membranes with deuterated CER NSd47, CER NPd47, and/or DFFA24 were examined. In Fig. 6A and B the δCH_2_ and δCD_2_ vibrations are provided measured at 10°C. Substituting either CER NS or CER NP with its deuterated counterpart (LPP:NSd47 and LPP:NPd47, respectively) resulted in a splitting distance of the δCH_2_ peaks of 9.3 ± 0.1 cm^−1^ (LPP:NSd47 model) and 9.5 ± 0.1 cm^−1^ (LPP:NPd47 model) (Table 3). A peak height ratio was calculated for the δCH_2_ peaks, to evaluate the difference of the average height of the two orthorhombic peaks (at 1,473 and 1,463 cm^−1^) and the peak height of the central peak (1,467 cm^−1^). Fitting these peaks and calculating their ratio allows a quantitative assessment of the chain interactions present in the model. The peak height ratio of the LPP:NPd47 composition is also different compared with the ratio in the spectrum of the protiated sample (Table 3) suggesting that protiated and deuterated chains are neighboring. However, the fittings did not lead to a statistically significant difference. A single peak is observed for the δCD_2_ vibrations at 1,088 cm^-1^ demonstrating that the deuterated lipids did not form separate lipid domains.

**Table 3:** Peak splitting values of the δCH_2_ and δCD_2_ vibrations for the six models, at 10°C, the peak height ratio of the δCH_2_ peaks (average of the peak height of the orthorhombic peaks at 1,473 and 1,463 cm^−1^ compared with the middle peak at 1,467 cm^−1^) and peak height ratio of the δCD_2_ (average peak height of the orthorhombic peaks at 1,086 and 1,091 cm^−1^ divided by the peak height of the middle peak at ∼1,088.5 cm^−1^). The values are shown as average ± SD of n ≥ 3 measurements

Lipid Model	δCH_2_ Peak split (cm^−1^)	δCD_2_ Peak split (cm^−1^)	δCH_2_ Peak Height Ratio (OR/MID)	δCD_2_ Peak Height Ratio (OR/MID)
LPP:prot	10.3 ± 0.1	-	3.1 ± 0.2	-
LPP:NSd47	9.3 ± 0.1	-	2.5 ± 0.1	-
LPP:NPd47	9.5 ± 0.1	-	2.4 ± 0.1	-
LPP:NSd47:DFFA24	-	5.2 ± 0.0	-	3.1 ± 0.2
LPP:NPd47:DFFA24	-	5.4 ± 0.1	-	3.5 ± 0.1
LPP:NSd47:NPd47:DFFA24	-	6.4 ± 0.1	-	5.0 ± 0.1

Next, FFA24 was replaced by DFFA24 alongside either CER NSd47 or CER NPd47 (Fig. 6A and B: LPP:NSd47:DFFA24 and LPP:NPd47:DFFA24 models, respectively). These two compositions displayed very different splitting of the scissoring vibrations in the spectra than observed for the LPP:NSd47 and LPP:NPd47 samples, and both compositions showed a similar trend. The δCH_2_ vibrations were characterized by a single peak at ∼1,468 cm^−1^ indicating that protiated hydrocarbon chains in orthorhombic packing are not neighboring. Two separated scissoring modes were observed in the δCD_2_ vibrations at 1,086 and 1,091 cm^−1^. The δCD_2_ peak to peak distance was 5.2 ± 0.0 cm^−1^ (LPP:NSd47:DFFA24 model) and 5.4 ± 0.1 cm^−1^ (LPP:NPd47:DFFA24 model) (Table 3). These results demonstrate that the deuterated acyl chains of the CER NSd47 or CER NPd47 were frequently neighboring the DFFA24 chains, as the introduction of DFFA24 allowed the coupling of the CD_2_-CD_2_ vibrations. To obtain quantitative information, the peak height ratio between the two orthorhombic scissoring modes (at 1,086 and 1,091 cm^−1^) and the central scissoring mode (at 1,088.5 cm^−1^) was calculated by peak fitting (Table 3). The statistical analysis results (*t* test with *P* <0.05) show that the peak height ratio for the LPP:NPd47:DFFA24 model is significantly higher than for the LPP:NSd47:DFFA24, illustrating that the CH_2_-CD_2_ chain interactions were less pronounced for the LPP:NPd47:DFFA24 than for the LPP:NSd47:DFFA24 model. Thus, in the LPP:NSd47:DFFA24 model the protiated and deuterated chains were more frequently neighboring compared with the LPP:NPd47:DFFA24 model.

Finally, when the majority of lipids were deuterated creating a model with DFFA24, NSd47, and NPd47, the FTIR spectrum displayed an increased δCD_2_ vibration peak splitting of 6.4 ± 0.1 cm^-1^, indicating a larger mean domain size of the deuterated chains in an orthorhombic packing (Table 3). The δCD_2_ vibration of the LPP:NSd47:NPd47:DFFA24 lipid sample had a deeper minima between the two peaks than in the δCD_2_ vibrations of the LPP:NSd47:DFFA24 and LPP:NPd47:DFFA24 models (Table 3). This suggests that the deuterated acyl chains of CER NS and CER NP were located in the same regions of the LPP unit demonstrating less CH_2_-CD_2_ interactions. The single peak observed in the δCH_2_ vibrations of this model indicates that the protiated lipid chains did not form phase separated domains and they were well mixed with the deuterated lipids or the remaining protiated lipids are partly in a hexagonal phase.

The neutron diffraction measurements were performed at 25°C; however, the lipid organization of these models is similar to that at 10°C, and these data for the δCH_2_ and δCD_2_ vibrations are shown in the supplemental Fig. S3.

## Discussion

**Fig. 7:**
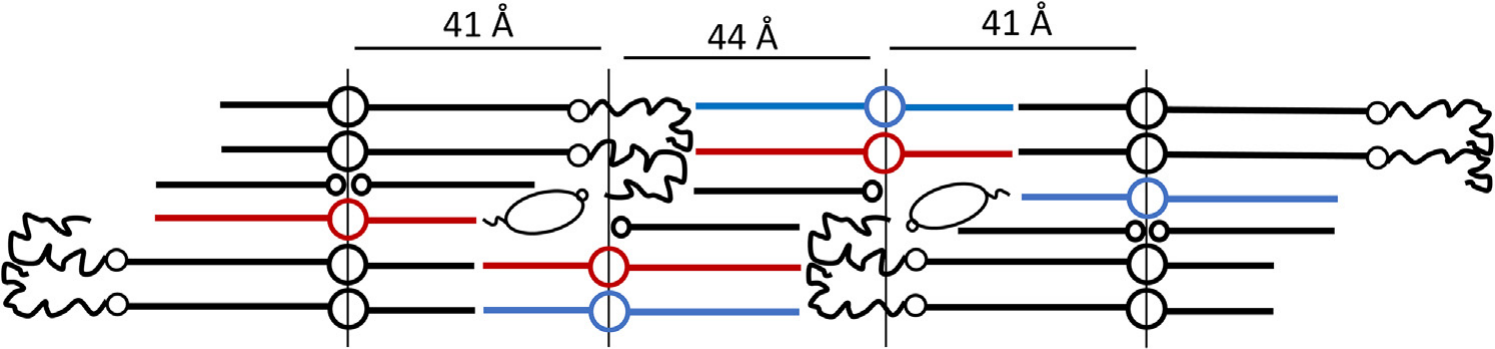
The molecular arrangement of the trilayer structure of the LPP unit according to the locations of CER NP and CER NS determined in this study and the positions of CER EOS, CHOL, and FFA C24 determined previously (40, 44). CER NS is depicted in red and CER NP in blue, while CER EOS, CHOL, and FFA C24 are drawn in black. The figure is adapted with permission from Mojumdar *et al.* (44).

In the present study a lipid model membrane consisting of CER EOS, CER NS, CER NP, CHOL, and FFA C24 forming the LPP was investigated. Previous studies revealed that the unit cell of the LPP has a three-layer structure (32, 40). In this study we were particularly interested in the location of CER NP in the LPP, as CER NP is the most abundant CER subclass in human SC (6, 7, 8, 10, 11, 30). This was compared with the position of CER NS, which is the most abundant CER subclass in mice, dog, and porcine SC (11, 27, 28, 29). Our results show that not only both the acyl chains of CER NP and of CER NS are positioned in the central layer but a minor part of the chains is also located in the outer layers of the LPP. Furthermore, the arrangement of the CER NP and CER NS positioned in the central layer is for both CERs primarily linear. Based on these results, we proposed the molecular arrangement of the lipids in the LPP unit, depicted in Fig. 7, adapted from the structure proposed by Mojumdar *et al.* (44).

### Arrangement of CER NP in unit cell of the LPP

Using neutron diffraction, six diffraction orders of the LPP could be detected, and we demonstrate a symmetric arrangement of the LPP unit cell, in agreement with previous studies that elucidated the electron density profile of the LPP trilayer using X-ray diffraction (32). The SLD profile of the LPP:NPd47 model revealed that most of the deuterated acyl chains of CER NP are located in the central layer, but a minor fraction is also positioned in the outer layers of the unit cell of the LPP (Fig. 7). The FTIR results for the LPP:NPd47:DFFA24 model demonstrate that the acyl chains of CER NPd47 and the DFFA24 chains are neighboring, as shown by the interactions of the deuterated chains and the δCD_2_ peak height ratio (OR/MID) for this composition. Thus, these chains are located in close proximity in the central layer of the unit cell.

The conformation of CER NP in an LPP model membrane has also not been examined before. Information on whether this lipid adopts a hairpin or an extended conformation can be obtained from the FTIR measurements. Focusing on the splitting of the δCD_2_ vibrations for the LPP:NPd47:DFFA24 and LPP:NSd47:NPd47:DFFA24 models, the inner layer of the LPP is characterized by large domains of deuterated chains. In a hairpin structure, the protiated CER NP phytosphingosine chains neighbor the deuterated acyl chains and this would strongly disturb the CD_2_-CD_2_ interactions in this layer and thus precludes a large splitting as encountered in the δCD_2_ vibrations. However, the opposite occurs, as when deuterated CER NP is used, the deuterated domain sizes increase. Therefore, it is very likely that CER NP adopts primarily an extended conformation with the phytosphingosine and acyl chains on either side of the headgroup.

### Arrangement of CER NP in comparison with CER NS in the LPP models

The SLD profile of the unit cell of the model membrane that includes CER NPd47 is similar to the model with CER NSd47; thus, not only the deuterated acyl chain of CER NS is located in the center of the LPP unit but also a minor fraction is located in the two boundary lipid layers. Assuming a C-C bond length of 0.125 nm, the length of the C24 acyl chain is approximately 2.9 nm (46). As the inner layer of the LPP has a total length of approximatively 4.4 nm, the acyl chains are interdigitated, as illustrated in the schematic arrangement proposed in Fig. 7. Interdigitating chains are quite often encountered in SC lipid organization, and it has been previously reported in SPP models (36, 49, 57, 58, 59) and LPP models (40, 44), as well as in computational models of the SC lipid models (60). All these studies support the interdigitated acyl chains and FFA chains in SC lamellar structures. In one of these papers, it has been discussed why interdigitation is more likely to occur to fit the chain length with the width of the central lipid layer than the alternative, namely, a large angle of the acyl and FFA chain with the basal plain of the lipid membrane (46). The results of this study are in agreement with the molecular organization proposed in earlier studies, which examined the localization of the acyl chain of CER NS both in a simple model comprising only CER EOS and CER NS and a complex lipid model mimicking the lipid composition of porcine SC (40, 46).

The difference between the structure of CER NP and CER NS is the C4-hydroxyl group located on the phytosphingosine chain, instead of the double bond of CER NS. This extra hydroxyl group can cause different lipid head group interactions. It was previously shown that the additional OH group of CER NP was very important in the formation of hydrogen bonds with surrounding hydrogen donors/acceptors (61), and CER NP caused 25% more H-bonds in a lipid bilayer, compared with CER NS (62). Moreover, it was hypothesized that the H-bonding could be participating in the connection of the ordered lipid domains in SC and decreasing the permeability of the SC, thus highlighting the importance of the CER NP subclass for the SC lipid matrix (61).

When comparing the δCD_2_ vibrations of the LPP:NPd47:DFFA24 and LPP:NSd47:DFFA24 models an interesting difference is noticed. The δCD_2_ peak height ratio (the two orthorhombic peaks resulting from the CD_2_-CD_2_ interactions) compared with the central peak (resulting from the CD_2_-CH_2_ interaction) for the LPP:NSd47:DFFA24 model is significantly lower than for the LPP:NPd47:DFFA24. This difference suggests that more CD_2_-CH_2_ interactions are encountered in the LPP:NSd47:DFFA24 model compared with the LPP:NPd47:DFFA24 model. This likely occurs in the outer layers of the LPP, where the protiated CER EOS and sphingoid chains are located and can interact with the deuterated acyl chains of the CERs. However, the neutron diffraction data indicate that the location of the two CERs is the same in the LPP trilayer. It might be that the δCD_2_ vibrations in the FTIR spectra are more sensitive to small differences in the localization of CER NS and CER NP.

### The arrangement of CER NS is not changed in lipid models of different complexity

CER NS is the most frequently investigated CER subclass with regard to its properties and molecular arrangement in model membranes mimicking the SC lipid composition. The arrangement of CER NS was also examined in our study, when analyzing the SLD profile of the LPP:NSd7 system. These results demonstrate the extended conformation of CER NS that is located in the inner layer of the LPP unit, as the acyl and sphingosine chains of CER NS are positioned on either side of the head group, as shown in Fig. 7. Based on the length of the sphingosine C18 chain, which extends over 15 C-C bonds from the head group, and assuming the length of the C-C bond of 0.125 nm, the total length of the sphingosine chain is approximatively 1.9 nm. Taking into account the location of the inner lipid head groups, the position of the terminally deuterated sphingosine chain of CER NS is calculated to be at 4.1 nm from the center, being close to the experimentally observed location.

Recent studies by Beddoes *et al.* provided insights into the conformation of CER NS both in a membrane prepared with a complex synthetic porcine CER mixture and a simple LPP lipid model system prepared with only two CER subclasses, CER EOS and NS (46, 63). In these studies, it was also concluded that CER NS with the head group in the inner head group layers adopts an extended conformation. Furthermore, in this paper it was thoroughly discussed why interdigitation occurs in the central layer of the LPP. An extended conformation of CER NS was also proposed in an SPP model composed of CER NS C24:CHOL:FFAC24, with an equimolar ratio of the lipids (64). This system was studied with ^2^H NMR and FTIR, and it was concluded that the acyl chain of CER NS was neighboring the FFAC24 chain and thus CER NS is present in a linear conformation in the SPP, similarly as observed in the LPP (46, 63).

### Lipid ordering in the outer layers of the LPP

When comparing the ν_s_CH_2_ peak positions of the LPP:prot and the LPP NSd47:NP47:DFFA24, the corresponding ν_s_CH_2_ frequencies indicate that the outer layers of the unit cell of the LPP, where CHOL, CER EOS, and the sphingoid chains of CER NS and CER NP are located, have a higher conformational disordering than the central region, where the acyl chains of CER NS and CER NP are primarily positioned. Furthermore, in the LPP:NSd47:NPd47:DFFA24 model the protonated lipid chains adopt at least partially an orthorhombic organization at 10°C, even though there is an increased ν_s_CH_2_ frequency. This can be concluded from the orthorhombic to hexagonal packing observed in the thermotropic behavior of ν_s_CH_2_ frequencies, indicating the phase transition occurs at 32–34°C. However, the single peak observed in the δCH_2_ vibrations of this model, attributed primarily to the protiated chains in the outer layers, indicates that a part of the protiated lipid chains may adopt a hexagonal packing. The presence of a hexagonal phase is also observed in the δCH_2_ vibrations of the LPP:prot model, which are characterized by two separated peaks and a small central peak, the latter indicating that at 10°C not all lipid chains are organized in an orthorhombic packing. Considering the proposed molecular organization of the LPP unit and the observations mentioned above, the sphingoid bases could be contributing to the formation of the hexagonal (less densely packed) phase, as it was suggested for an SPP model with CER NS (64).

### Extrapolation of the results to the lipid matrix in SC

Understanding the arrangement of the barrier lipids within the SC is important for advancing the knowledge on the skin barrier function. The results of this study contribute to a better understanding of the SC lipid organization. First of all, CER NS and CER NP are positioned at the same location in the unit cell of the LPP and both CER subclasses are primarily aligned in a linear arrangement, in which the acyl chains are interdigitating together with the FFA chains. This linear arrangement has various advantages for the SC to provide an excellent barrier. Owing to the reduced cross section per lipid molecule in a linear arrangement compared with the hairpin structure, the linear arrangement accommodates a tighter packing of the hydrocarbon chains in the structure, which is favorable for the barrier functionality (65).

Furthermore, a linear arrangement makes the structure more flexible as the interdigitating chain length can be adapted to the requirements to obtain a dense and thermodynamically stable structure. A linear conformation of CER NS and CER NP also provides a tight connection of the adjacent lipid layers, so it reduces the permeability through the SC lipid matrix (65) and it discourages swelling of the lipid lamellae during hydration (19). In this respect it is important to mention that a linear arrangement has been proposed not only in the LPP but also in the SPP unit cell with CER NS (64). Interestingly, in both the LPP and SPP unit structure, the CHOL is at a similar position as the sphingoid base of the CERs, while the FFA chains and acyl chains are neighboring. A linear arrangement has also been proposed for the molecular models proposed by Norlén *et al.* (66).

Another important message is the similar position of the acyl chains of both CER NP and CER NS in the LPP unit as it indicates the adaptability of the lamellar phase to differences in the lipid head group architecture. This observation is a possible explanation that, while in human SC CER NP is one of the most prevalent CER subclasses, in mice, porcine and dog SC CER NS is the most abundant one, the SC lipid organization is very similar in all species. However, the effect of changing the concentration of CER NS and CER NP on their position and alignment in the unit cell of the LPP has not yet been investigated and may affect the outcome.

Using Ruthenium tetroxide (RuO_4_) staining the lamellar organization in SC was for the first time reported by Madison *et al.* (67). These studies showed broad-narrow-broad sequence of lucent bands in one repeating unit. RuO_4_ is a strong oxidizer that reacts with the double bonds of the lipid chains and the hydroxyl groups of the CERs (68). Thus, both the head groups and the linoleate position will turn into dark regions in the electron micrographs. As the CER EOS linoleate chains are present in the inner part of the central region close to the inner head group region, this inner region represents the narrow lucent band, while the two broad lucent bands represent the two outer regions. Therefore, our proposed molecular arrangement is in agreement with the RuO_4_ pattern reported.

### Selection of the CER composition in the lipid systems

As the composition of the human SC lipid matrix is too complex to selectively analyze the localization of individual CER subclasses in the LPP structure, model systems composed of selected lipids can be used. Previous studies show that lipid models provide reproducible structures mimicking those found in SC of native skin. These lipid models consisted of CERs extracted from native porcine or human SC (29, 32) or a synthetic CER composition that mimicked the CER composition of the porcine SC (31, 32, 69). However, to obtain more detailed information about the molecular arrangement, a simple model with a limited number of lipids is required. The advantage of a simple model is the possibility to incorporate sufficient deuterated CERs of interest to provide information about the interactions between different lipid subclasses, thus we chose such a model for studying the localization of CER NP and CER NS in the LPP unit.

An important difference between the native SC lipid composition and the synthetic lipid models is the concentration of CER EOS. This CER is crucial for the formation of the LPP, and varying its concentration leads to simultaneous formation of an LPP and SPP phase at 15 mol% (32, 39, 41), the approximate level in human SC, while a minimum of 30 mol% CER EOS is required for the formation of only the LPP (41, 70). Increasing the concentration of CER EOS to 40 mol% did not change the LPP structure and organization and resulted in a peak separation of crystalline CHOL and the fourth order diffraction peak of the LPP, important for analyzing neutron data (42).

## Conclusion

In this study we determined for the first time the position of the acyl chain of CER NP in the LPP unit cell of a simple lipid model membrane. Our studies revealed that, in the 12.6 nm repeating unit of the LPP, the acyl chain of CER NP is predominantly located in the central part of the trilayer structure, with a minor fraction present at the unit cell boundary. The location and the arrangement of CER NP was similar to that of CER NS and both CERs adopt a linear arrangement, indicating that, even though there is a difference in the headgroup structure of CER NS and CER NP, they adopt a similar molecular arrangement in the LPP unit. This study expands the current knowledge of the molecular arrangement of lipids in the LPP, a very important structure present in the native SC.

## Data availability statement

All data are included in the article and supporting information.

## Supplemental data

This article contains supplemental data.

## Conflict of interest

The authors declare that they have no conflict of interest with the contents of this article.
