# Supplementary material for: Phytosphingosine ceramide mainly localizes in the central layer of the unique lamellar phase of skin lipid model systems
Source: J Lipid Res. 2022 Aug 2;63(9):100258. doi: 10.1016/j.jlr.2022.100258 (PMC9421324; doi:10.1016/j.jlr.2022.100258)
Supplement: Supplemental Data [file mmc1.docx]

**SUPPLEMENTAL INFORMATION:**

**Phytosphingosine ceramide mainly localizes in the central layer of the unique**

**lamellar phase of skin lipid model systems**

**Andreea Nădăban^1^, Gerrit S. Gooris^1^, Charlotte M. Beddoes^1^, Robert M. Dalgliesh^2^, Joke A. Bouwstra^1*^**

**^1^ Division of BioTherapeutics, Leiden Academic Centre for Drug Research, Leiden University, Leiden, The Netherlands**

**^2^​ ISIS Neutron and Muon Source, Science and Technology Facilities Council, Rutherford Appleton Laboratory, Didcot, United Kingdom**


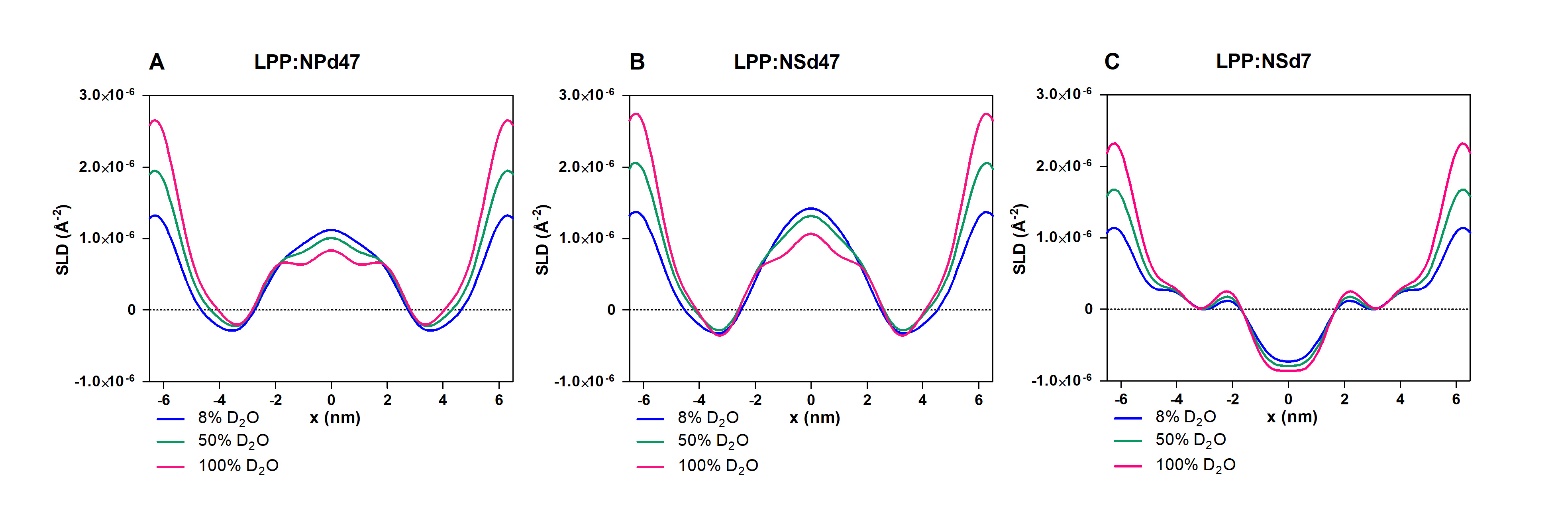


**Supplemental Figure S1**. The SLD profiles for the LPP:NPd47 (A), LPP:NSd47 (B) and LPP:NSd7 (C) models plotted for each of the three D_2_O/H_2_O buffer hydration levels (8% in blue, 50% in green, 100% in magenta).





**Supplemental Figure S2.** Thermotropic curves of the stretching vibrations for the partially deuterated systems: (A) LPP:NSd47:DFFA24 and (B) LPP:NPd47:DFFA24. The phase transitions temperatures of the lipids are plotted as a function of the ν_s_CH_2_ and ν_s_CD_2_ peak position, on the left and right y-axis, respectively. Both the protiated (blue circle) and deuterated (red square) lipids melted over the same temperature range, indicating that the deuterated lipids were integrated with the protiated lipids in the lipid system.


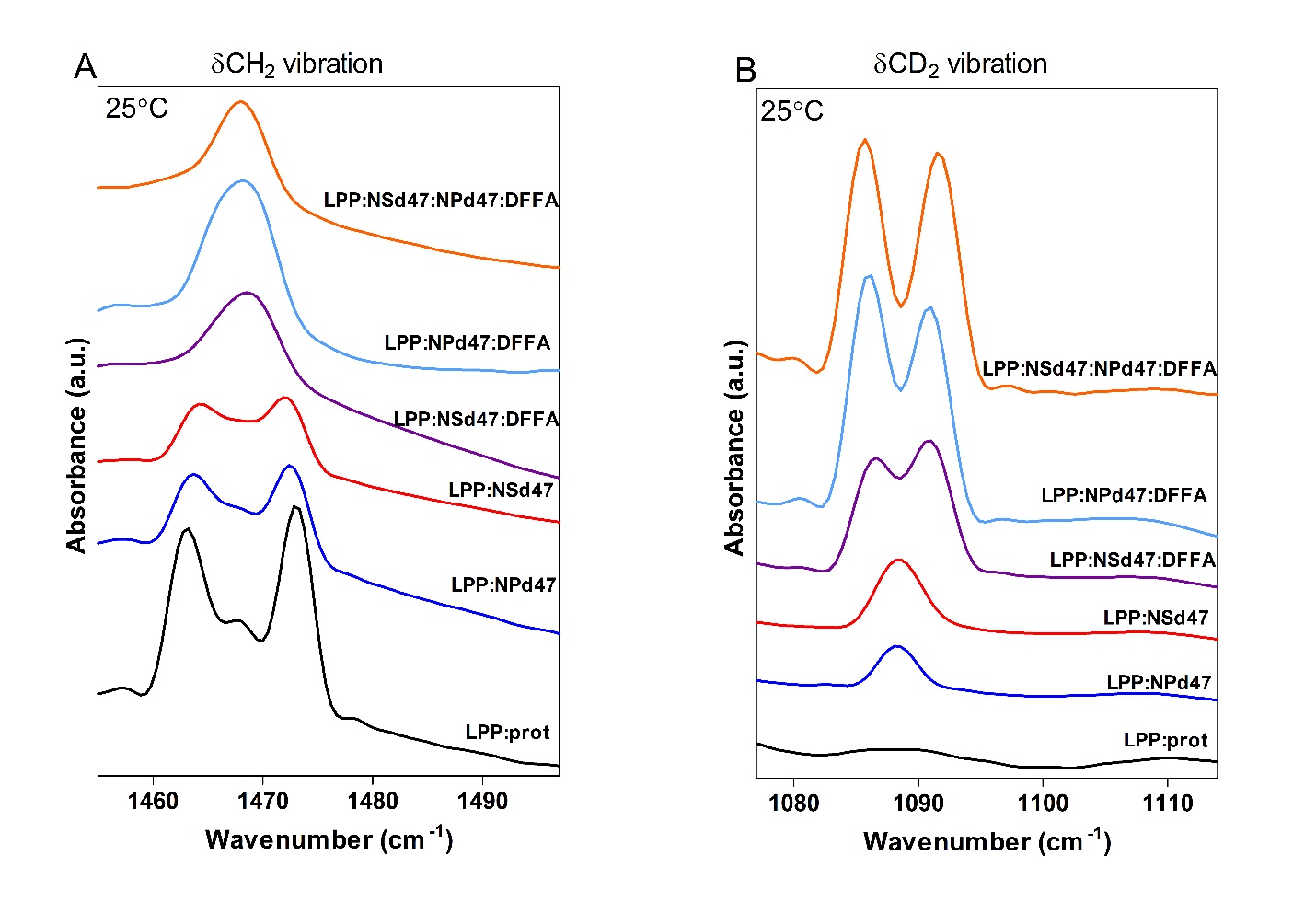


**Supplemental Figure S3.** The δCH_2_ (A) and δCD_2_ (B) vibrations of the six lipid systems, determined by FTIR, at 25°C (same temperature used during the neutron diffraction measurements). Each curve has an annotation with the lipid model it represents.
